# Draft Genome Sequences of *Saccharibacter* sp. Strains 3.A.1 and M18 Isolated from Honey and a Honey Bee (*Apis mellifera*) Stomach

**DOI:** 10.1128/genomeA.00744-17

**Published:** 2017-07-27

**Authors:** Alexandra Veress, Tímea Wilk, János Kiss, Ferenc Olasz, Péter P. Papp

**Affiliations:** Agricultural Biotechnology Institute of the National Agricultural Research and Innovation Centre, Gödöllő, Hungary

## Abstract

The annotated draft genome sequences of two recent *Saccharibacter* sp. strains isolated from honey and a honey bee stomach in 2014 are reported here. Currently, two *Saccharibacter* whole-genome sequences are available in databases; thus, the sequences of our new isolates will contribute to a better understanding of *Saccharibacter* genomes.

## GENOME ANNOUNCEMENT

*Saccharibacter* species (*Acetobacteraceae*) are aerobic Gram-negative bacteria that often occur in sugar-rich environments, for instance, in the gut of sugar-feeding insects (1–4), and they are suggested to be symbionts of insects. Despite the possible importance of these species, only limited information is available about their genome organization. Surprisingly, only two *Saccharibacter* genome sequences can be found in databases to date (*Saccharibacter* sp. strain AM169, accession number CBLY01 [1], and *Saccharibacter floricola* DSM15669, accession number ARJS01 [5]). Here, we report draft genome sequences of *Saccharibacter* sp. strain 3.A.1 isolated from honey and *Saccharibacter* sp. strain M18 isolated from a honey bee (*Apis mellifera*) stomach. The samples were derived from different apiaries located in the central region of Hungary.

In order to investigate the genomes of *Saccharibacter* sp. strains 3.A.1 and M18, total DNA was isolated, and 600- to 630-bp fragment libraries were prepared by UD GenoMED (Debrecen, Hungary). The 2 × 300-bp Illumina paired-end genome sequencing was performed by the University of Szeged, Department of Biochemistry and Molecular Biology (Szeged, Hungary) as a custom service using Illumina’s MiSeq platform. The numbers of reads were 810,000 for *Saccharibacter* sp. 3.A.1 and 3.1 million for *Saccharibacter* sp. M18. The estimated coverages of the whole genomes were 120× and 450×, respectively.

The reads were *de novo* assembled using A5-miseq (6). The total lengths of the chromosomal contigs for *Saccharibacter* sp. 3.A.1 and M18 were 2,023,510 and 2,086,874 bp, and their GC contents were 49.15% and 52.73%, respectively. The assembled genome sequences were annotated using the RAST annotation server (7). We set the genetic code to 11 (*Archaea*, *Bacteria*). In the whole genomes of *Saccharibacter* sp. strains 3.A.1 and M18, 1,913 and 2,001 annotated genes, 100 and 102 tRNAs, and 13 and 15 rRNAs were identified, respectively.

Pairwise comparison (8) of the sequences revealed a striking similarity (98.69%) between the two strains. The genome sequence of *Saccharibacter* sp. 3.A.1 proved to be almost identical (99.24%) to that of *Saccharibacter* sp. AM169 and 77.20% similar to that of *S. floricola* DSM15669, while *Saccharibacter* sp. M18 showed 98.72% and 77.81% similarity to those strains, respectively.

The draft sequence of *Saccharibacter* sp. M18 contains additional scaffolds that cannot be aligned to that of *Saccharibacter* sp. 3.A.1. Further analysis of these scaffolds revealed the presence of 13,145-bp and a 7,488-bp plasmids in *Saccharibacter* sp. M18 without significant sequence homology to hitherto-known plasmids.

The 16S rRNA gene sequences previously suggested that strains 3.A.1 and M18 can be classified as *Saccharibacter* spp., which was confirmed by the analyses of six further genes (*gyrA*, *gyrB*, *dnaJ*, *recA*, *rnaP*, and *groEL*). Our draft genome sequences may offer better insight into the origin and evolution of this lesser-known group of bacteria.

### Accession number(s).

The draft genome sequences of *Saccharibacter* sp. strains 3.A.1 and M18 genomes have been deposited in the NCBI GenBank database under the accession numbers MNPT00000000 and MNPS00000000, respectively.
